# Determinants of overweight and/or obesity among school adolescents in Butajira Town, Southern Ethiopia. A case-control study

**DOI:** 10.1371/journal.pone.0270628

**Published:** 2022-06-28

**Authors:** Shemsu Kedir, Kalkidan Hassen, Yabsra Melaku, Musa Jemal

**Affiliations:** 1 Departement of Public Health, College of Medicine and Health Science, Werabe University, Werabe, Ethiopia; 2 Department of Nutrition and Dietetics, College of Health Science, Jimma University, Jimma, Ethiopia; PLOS ONE, UNITED KINGDOM

## Abstract

**Background:**

The global rise of adolescent overweight and obesity is posing a new challenge to the public health sector by determining the forthcoming generation for the most awful and upsetting quality of social life by inducing bantering, social isolation, and stigmatization among children that contribute to the mental wellbeing of the growing adolescents. Risk factors for overweight and/or obesity might not be the same across different regions due to differences in socioeconomic characteristics, culture, ethnicity, and geographical location. Moreover, in this study area, no report has been documented so far on the determinant factors of overweight and/or obesity among school adolescents. Hence, this study aimed at identifying context-specific determinants of overweight and/or obesity among adolescents in the study area.

**Methods and materials:**

School-based unmatched case-control study design was employed from March 1–30, 2019, in Butajira town, Southern Ethiopia. Data were collected regarding sociodemographic, dietary practice, physical activity, nutritional knowledge-related factors, and anthropometric measurements. Multivariable logistic regression models were fitted to identify independent predictors of overweight/obesity.

**Results:**

We enrolled 297 adolescents: 99 cases, 198 controls. Multivariable binary logistic regression analysis revealed that those in high socioeconomic status [AOR = 5.8, 95% CI (2.66, 12.5)], consumed soft drinks 3 and above times per week [AOR = 3.7, 95% CI (1.8, 7.3)], physically inactive [AOR = 4.4 95% CI (1.68, 11.6)], spent free time by watching television/movies for 3 and above hours per day [AOR = 8.6, 95% CI (4.3, 17)] and with poor nutritional knowledge [AOR = 3.4, 95%CI (1.7, 6.9)] were significantly associated with overweight and/obesity.

**Conclusion:**

High socioeconomic status, consumption of soft drinks, physical inactivity, sedentary behavior, and poor nutritional knowledge were significantly associated with overweight/obesity. Therefore, strengthening parent and school-based health education in healthy nutrition behaviors and promotion tactics such as enhancing physical activity, limiting watching television, and soft drinks will be helpful to minimize overweight and obesity among adolescents.

## Background

Overweight and obesity are defined by the World Health Organization (WHO) as an excess of body fat that may impair or hinder health [1]. Overweight and obesity are both long-lasting conditions that are the consequence of an energy imbalance over a period, that arise when the number of calories expended is not equal to the number of calories used by the body. The cause of this energy imbalance can be due to a combination of different factors and varies from one person to another [2]. The WHO defines adolescence as the period of life transition from 10 to 19 years [3].

Worldwide, overweight and obesity are becoming one of the most challenging health concerns with the worrisome rise in children and adolescents. The prevalence of overweight and obesity among children and adolescents aged 5–19 has escalated radically from just 4% in 1975 to over 18% in 2016. The rise has occurred similarly among both boys and girls: in 2016 18% of girls and 19% of boys were overweight [1]. Around 55% of obese children go on to be obese in adolescence, around 80% of obese adolescents will still be obese in adulthood, and around 70% will be obese over age 30 [4]. Overweight and obesity are the fifth leading cause of global death. As estimated by WHO, at least 2.8 million adults die each year because of being overweight /obese. In addition, 44% of the diabetes burden, 23% of the ischemic heart disease burden, and between7% and 41% of certain cancer burdens are attributable to overweight and obesity [2].

According to (WHO) report in 2016, an estimated 41 million deaths occurred due to non-communicable diseases (NCDs), accounting for 71% of the overall total of 57 million deaths [5]. In Africa, under-nutrition is the major nutritional problem affecting both children and adolescents. However, overweight and obesity were noticeably high with a prevalence of 8.5% in 2010 and predicted to be 12.7% by 2020. This situation pinpoints a double burden of malnutrition and epidemiological as well as nutrition transition by several socioeconomic and demographic changes [2]. Currently, about 20–50% of the urban population in Africa is classified as either overweight or obese. Moreover, it has been estimated that, by 2025, 3/4th of the obese population worldwide will be in non-industrialized countries [6].

The combined prevalence of overweight and obesity among school adolescents in Addis Ababa and Jimma towns were 8.5% and 13.3%, respectively [7, 8]. According to the study conducted in Hawassa, high school adolescents showed that the combined prevalence of overweight and /or obesity was 15.6% [9]. These findings showed that overweight and obesity are becoming the health problems of developing countries including Ethiopia.

Forces of globalization have led to creeping monotonous diets and an increased intake of energy-dense foods that are high in fat; an increase in sedentary behavior as well as physical inactivity due to changing modes of transportation, and increasing urbanization [1].

Ethiopian House of Peoples Representatives endorsed a law restricting smoking in public areas and prohibiting alcohol commercials on public media outlets, 2019. National Nutrition Program from 2016–2020 suggested timely interventions will help prevent NCDs or reduce their severity and consequences [10]. Even though the adolescent nutrition problem in Ethiopia is becoming a focus but still there is no strong action being taken. The national prevalence of overweight and/or obesity among adolescents is not established so far [11].

Although there are cross-sectional studies that have been conducted in different parts of Ethiopia on the prevalence of overweight and obesity among adolescents, few studies have been conducted regarding its determinants using a case-control study design. These few studies did not consider the most important variables associated with obesity like household assets, nutritional knowledge, and were methodologically conducted only in private schools [12]. In addition to this, risk factors for overweight and/or obesity might not be the same across different regions due to differences in socioeconomic characteristics, culture, ethnicity, and geographical location. Moreover, in Southern Ethiopia, no report has been documented so far on the determinant factors of overweight and/or obesity among school adolescents. This study aimed to address the aforementioned gaps and contextual specific determinants of overweight and obesity as a critical undertaking to formulate preventive programs.

## Methods and materials

### Study design and setting

The study was conducted in Butajira Town, Gurage zone, Southern Nation, Nationalities, and Peoples of Representatives. Butajira town is located 132 km to the south of Addis Ababa. The town has an area of 32. 43 square kilometers with an altitude of 2131 meters above sea level. Butajira Town is a growing city in Ethiopia with an estimated population of about 52,228. According to the 2018/2019 town’s education office statistics, six (06) governmental (6646 students) and 05 (945 students) private schools are found in the town. There are 7591 adolescents of which 3928 students were male in both categories of schools. The study area is also easy to access in travel within 30 minutes and the study populations are well cooperating and stable. The study was conducted from March 1 to March 30 /2019. A school-based unmatched case-control study design was employed. The study population was randomly selected schools in private and governmental schools that fulfill inclusion criteria and who met the definition of overweight/obesity: BMI-for-age Z scores above >1SD and Control/Normal: BMI-for–age Z scores between −2SD < BAZ ≤1SDA.

### Sample size determination

The sample size was determined using Epi Info7 preview version 3.5.3 using Stat- Calc by taking the main determinants of overweight and obesity from a previous study [12, 13]. Considering, the proportion of sedentary activities four hour and above was 40% among control, exposed, and AOR 2:1. Control to case ratio = 2:1 by using Kelsey. By considering the 5% nonresponse rate, the sample size increases to 297. Then 99 cases and 198 controls were enrolled in the study. Butajira Town school was stratified into governmental and private. There are 5 private and 6 governmental schools. From both governmental and private schools, 2 schools from each were selected randomly by Microsoft Office Excel. To identify adolescents that have been eligible for cases and controls, Census was conducted in selected schools on 2691 adolescent students two weeks before data collection and among this, 179 cases and 1826 controls were identified. Proportionally allocated sample sizes for both case and controls were selected by simple random sampling using excel generated random numbers after the sampling frame was developed for cases and controls separately for private and governmental schools.

### Data collection

First of all, the questionnaire was translated into Amharic language and then back-translated to English by independent language experts for their consistency. A total of two supervisors and four data collectors were recruited for data collection and a census was performed to identify cases and controls before two weeks. The participants aged from 10–19 years were classified as cases and controls depending on their BAZ status and data were collected among selected cases and controls randomly based on the sampling frame. Adolescents with body deformities like kyphosis and scoliosis, individuals who were severely sick, unable to communicate and hear, and thinness (BMI-for–age Z scores ≤ −2SD) were excluded from the study.

### Measurements and operational definition

Data were collected by face-to-face interview method using a semi-structured questionnaire which is adopted from WHO STEP-WISE chronic disease, Food Agriculture Organization, and other kinds of literature [14, 15] and taking anthropometric measurements.

Weight was measured using a portable standing (Seca Germany) scale from 0 to 150 kg and recorded to the adjacent of 0.1 kg. The height was measured by a portable Stadiometer and recorded to the nearest 0.1cm. It was calibrated against known weight regularly. On the pretest, 2.7% and 1% of the coefficient of variation was determined for their weight and height respectively. At the time of measurements standardization exercise was performed like the subjects wore light clothes, measured against the wall without footwear and with heels together, shoulder blades, buttocks, and calf touching measurement board and their heads positioned and eyes viewing straight ahead so that the line of sight was perpendicular to the body to minimize the technical error of measurements. To minimize anthropometric measurement variability we used the same measurer for the whole data collection. Two readings of height and weight were taken and their means were considered as final. Overweight and obesity of the study subjects were determined by the new WHO reference data using the WHO Anthro-Plus computer program. The values between 1SD to 2SD for specific age and sex were considered overweight and values above 2SD for age and sex are considered as obesity [16].

Dietary diversity score [DDS] was assessed using a food frequency questionnaire containing 28 food items that are commonly consumed in the study area. The list of food items was developed based on a wide-ranging interview of key informants from the study area who knew the culture and language as well as commonly consumed. The food frequency questionnaire was refined based on the result of the pretest on 14 adolescents’ responses by observing patterns of weekdays of common food consumption. Cronbach’s alpha result was 0.79 during the pretest. Adolescents were implied as ‘‘consumers” of a food item if they had consumed the food item at least once per week. As there is no Ethiopian classification of food groups, the 28 food items of the food frequency questionnaire were grouped into nine groups according to Women Dietary Diversity Score (WDDS) [17]. For instance, an adolescent who consumed one item from each of the food groups at least once during the week would have the maximum DDS 9, and those who did not consume per week score 0 for all food groups [18].

The Global Physical Activity Questionnaire (GPAQ) developed by WHO for physical activity surveillance was used to collect information on physical activity participation in three settings (or domains) including activity at labor, mobile to and from places, leisure activities, and sedentary behavior. Data collectors were asked participants about physical activity level daily in a minute and then changed to weekly and determine whether active or not. Data collectors were asked about sedentary activities spent hours per day. The activity level of the study participants was evaluated according to the standard total physical activity calculation guideline. Those who score below 600 Metabolic Equivalent Time (MET) are considered as physically inactive, between 600 and 1500 MET considered as moderate physically active, and above 1500 MET considered as vigorous physically active [15].

Nutritional Knowledge Index: Thirteen questionnaires were adapted and checked their reliability with a Cronbach’s alpha value of 0.73 from the Food and Agriculture Organization of United Nations for school children. Based on this question, the nutritional knowledge index variable was created using principal component analysis and categorized into two groups based on the mean value. Thus, the first component students scored “poor knowledge” and the second component students scored “good knowledge” [14].

Wealth index: The 2016 Ethiopian demographic Health Survey tool for wealth index was used and the pretest Cronbach’s alpha result was 0.78. All study participants were asked about the ownership of fixed assets in their household with a score of 1 given to those who own the asset and a score of “0” given to those who did not own it. Then principal component analysis was used to develop the wealth index and categorize it into 3 tertiles [11].

Adolescent Age Classification: The age was classified in to early, middle, and late adolescent. WHO classifies adolescence as early (10–13 years), middle (14–16 years) and late adolescence (17 and above years).

Overweight and/or Obese: BMI-for-age Z scores >1SD [19] and

Normal: BMI-for–age Z scores between −2SD < BAZ ≤ +1SD [19].

### Data processing and statistical analysis

Data were checked for completeness and consistency and then edited, coded, and entered using Epi-data version 3.1, and then exported to SPSS version 21 for further analysis. Variables were checked for missing values before analysis. Descriptive statistics had been calculated for all variables following their kind. Means, medians, and standard deviations had been computed for continuous variables. The categorical variables are presented by frequencies with proportion. Principal Component Analysis was employed for both wealth index and nutritional knowledge. As well, its assumptions like sample size, the ratio of variables to cases, the variables included were dichotomous, the measure of sampling adequacy (KMO and Anti-Image ≥0.5), Bartlett test of sphericity were statistically significant(P<0.05). In addition to this, no complex structures were seen and explained variation was also satisfied. Bivariate analyses were done to identify the candidate variables with a p-value ≤ 0.25 [20] for a multivariable logistic regression model to determine risk factors of overweight and/or obesity. The multivariable binary logistic regression had good model fitness by using the Hosmer-Lemeshow model test with Pvalue = 0.69 and x^2^ = 7. Multicollinearity of the independent variables was checked by the variance inflation factor which was (VIF) <5 for all candidate variables. All tests were two-sided and p < 0.05 with a 95% confidence interval were considered statistically significant.

### Data quality control

The one-day training was given for all data collectors, two Health officers as a supervisor, and four Diploma Nurses as data collectors, about the objectives, process of data collection. Demonstration of interview thoroughly and taking measurements were given for each trainee to reduce inter-observer error. The instrument was pretested by 5% (14 participants) out of the selected school’s participants for clarity, understandability, flow, and construction, and those questions found to be unclear or confusing were modified based on the result of the pretest.

The weight scale was calibrated at 0 with no object on it and placed on the level surface before measurements were carried out. Every morning and when the instruments move apart, calibration and validation have checked the scales by 2kg metal iron sheet to keep their reliability. The supervisors were supervised and reviewed every questionnaire for completeness and logical consistency and corrections are made. Data collectors submitted the collected data daily to supervisors and the principal investigator. Each questionnaire was checked daily before data entry for completeness and consistency. Data coding, entry, and cleaning were performed by the principal investigator. The principal investigator collected the completed questionnaires every day and was responsible for the coordination and on-spot supervision of the overall data collection process.

### Ethical approval and consent to participate

Ethical clearance was obtained from the Institute Review Board (IRB) of Jimma University (Ref.No. JHRPGD/690/19). There are no potential risks that may cause any harm to study participants in any form. Then the support letter was obtained from the Butajira Education office and sent a support letter for each sub-city. Finally, each sub-city sent the support letter for each selected high school in that sub-city. Then nature of the study was fully explained to each school administration. After getting permission from the school to participate in the study, written consent was taken from students’ families by sending consent letters to adolescents and verbal assent was taken from each participant. Coding and aggregate reporting were used to eliminate names and other personal identification of respondents throughout the study process to ensure anonymity, privacy, and confidentiality. For those obese adolescents, mass advice about a healthy diet and physical activity was given in selected schools.

## Results

### Socio-demographic characteristics of study participants

A total of 297 adolescents from both government [209(70.4%)] and private [88(29.6%)] were involved in this study with a response rate of 100%. Of these, 62.6% of cases and 55.6% of controls were females. The mean age of adolescents was 14.13±2.33. 49.5% of cases and 47% of controls were early adolescence between the age of 10–13 years old. 56.6% of cases and 20.7% of controls were in the higher wealth index category. Whereas, 17.2% of cases and 44.4% of controls were in the low wealth index category. Regarding father occupation, 57.6% of cases and 47% of controls were merchants. Regarding maternal education, 19.2% of cases and 26.8% of controls were no formal education. The mean family size of adolescents was 3.9±1 (Table 1).

**Table 1:** Distribution of socio-demographic characteristics of overweight and/or obesity among school adolescents in Butajira Town, Southern Ethiopia, 2019.

Variables	Category	Cases	Controls
		N(%)	N(%)
	Male	37 (37.4%)	88 (44.4%)
Sex	Female	62 (62.6%)	110 (55.6%)
Maternal education	Not read and write	19 (19.2%)	53 (26.8%)
	Formal education	80 (80.8%)	145 (73.2%)
Paternal education	Not read and write	19 (19.2%)	42 (21.2%)
	Formal education	80 (80.8%)	156 (78.8%)
Family size	<4 family	49 (49.5%)	83 (41.9%)
	≥4 family	50 (50.5%)	115 (58.1%)
School type	Governmental	71 (71.7%)	138 (69.7%)
	Private	28 (28.3%)	60 (30.3%)
Age category (year)	Early adolescence	49 (49.5%)	93 (47%)
	Middle adolescence	34 (34.3%)	72 (36.4%)
	Late adolescence	16 (16.2%)	33 (16.7%)
Wealth index	High	56 (56.6%)	41 (20.7%)
	Medium	26 (26.3%)	69 (34.8%)
	Low	17 (17.2%)	88 (44.4%)

### Characteristics of dietary practices and related factors

The mean individual dietary diversity of adolescents were 3.66±1.89. 42(42.4%) of the cases and 102(51.5%) of controls had a history of taking fast foods. 67.7% of cases and 45.5% of controls were consuming snacks. Low dietary diversity scores were reported from 67.7% of cases and 45.5% of controls. Moreover, 77.8% of cases and 51% of controls were the habits of consuming soft drink habits ≥3 times per week (Table 2).

**Table 2:** Distribution of dietary-related factors of overweight and/or obesity among school adolescents in Butajira Town, southern Ethiopia, 2019.

Variables	Category	Cases N(%)	Control N(%)
fast food	Yes	42 (42.4%)	102 (51.5%)
	No	57 (57.6%)	96 (48.5%)
Snack	Yes	67 (67.7%)	90 (45.5%)
	No	32 (32.3%)	108 (54.5)
Regular meals	1–2 times/day	30 (30.3%)	72 (36.4%)
	3–4 times/day	69 (69.7%)	126 (63.6%)
Skip meals	No	36 (36.4%)	74 (37.4%)
	Yes	63 (63.6%)	124 (62.6%)
Soft drinks per week	≥3x/week	77 (77.8%)	101 (51%)
	<3x/week	22 (22.2%)	97 (49%)
Category of IDDS*	Low	67 (67.7%)	89 (44.9%)
	High	32 (32.3%)	109 (55.1%)

*IDDS: Individual Dietary Diversity Score

### Characteristics of physical, sedentary behaviors, recreational activities, and nutritional knowledge

Regarding physical activity, 71.7% of cases and 44.9% of controls were physically inactive. Whereas, 8.1% of cases and 22.7% of controls were in vigorous physical activity. 48.5% of cases and 57.1% of controls use vehicles for transportation. Moreover, 52.5% of cases and 12.6% of controls have sedentary behaviors ≥3 hours per day. Regarding nutritional knowledge, 77.8% of cases and 60.6% of controls had poor knowledge (Table 3).

**Table 3:** Distribution of physical, sedentary behaviors, recreational activities, and nutritional knowledge related factors of overweight and/or obesity among school adolescents in Butajira Town, Southern Ethiopia, 2019.

Variables	Category	Cases	Controls
		N(%)	N(%)
Physical Activity	Inactive	71 (71.7%)	89 (44.9%)
	Moderate	20 (20.2%)	64 (36.3%)
	Vigorous	8 (8.1%)	45 (22.7%)
Transportation in vehicles	Yes	48 (48.5%)	113 (57.1%)
	No	51 (51.5%)	85 (42.9%)
Sedentary behavior	≥3 hours/day	52 (52.5%)	25 (12.6%)
	<3 hours/day	47 (47.5%)	173 (87.4%)
Nutritional knowledge	Good	22 (22.2%)	78 (39.4%)
	Poor	77 (77.8%)	120 (60.6%)

### Bivariable logistic regression for candidate variables for overweight and obesity among school adolescents in Butajira Town, Southern Ethiopia, 2019

Thirteen Candidate variables were drawn from bivariate logistic regression analyzed each predictor with a dependent variable from socio-demographic (Female adolescents, paternal occupation, maternal education, low family size and high wealth index), from dietary practice (Snack, fast food, and soft drink consumers and low IDDS), from physical and recreational activities (Physical inactivity, mode of transportation in vehicles and sedentary behaviors≥3 hours/day) and Nutritional Knowledge.

### Determinants of overweight and obesity among school adolescents in Butajira Town, Southern Ethiopia, 2019

The odds of overweight and obesity among adolescents from the high wealth quantiles were 5.8 times more as compared to adolescents from the low wealth quantiles [AOR = 5.8 (95% CI: 2.66,12.5)]. Likewise, the likelihood of adolescents being physically inactive was 4.4 times more for overweight and obesity as compared to Normal adolescents [AOR = 4.4(95% CI: 1.68,11.6)]. Regarding sedentary behavior, the likelihood of overweight and/or obesity among adolescents who spent free time watching television/movies for 3 and above hours per day was 8.6 times more than adolescents from their counterparts [AOR = 8.6(95% CI: 4.3,17)]. Adolescents who were consuming soft drinks 3 and above times per week were 3.7 times more likely to be overweight and/or obese as compared to adolescents who consumed soft drinks less than 3 times per week [AOR = 3.7 (95% CI:1.8,7.3)]. Moreover, the odds of overweight and/or obesity among adolescents who had poor knowledge in nutrition was 3.4 times higher than adolescents who had good knowledge in nutrition [AOR = 3.4(1.7,6.9)] (Table 4).

**Table 4:** Multivariable logistic regression analysis of factors associated with overweight/obesity among school adolescents in Butajira Town Southern Ethiopia, 2019.

Variable	category	Overweight and obesity		COR^^^ with 95% CI	AOR^#^ with 95% CI
		Yes, N (%)	No N(%)		
	High	56 (56.6%)	41 (20.7%)	7 (3.6,13.6)	5.8 (2.66, 12.5)**
Wealth	Medium	26 (26.3%)	69 (34.8%)	1.95 (0.98,3.8)	1.79 (0.87, 4.47)
index	Low	17 (17.2%)	88 (44.4%)	1	1
	No	71 (71.7%)	89 (44.9%)	4.5 (1.9,10)	4.4 (1.68, 11.6)*
Physical activity	Moderate	20 (20.2%)	64 (36.3%)	1.75 (0.7,4.3)	2.2 (0.75, 6.3)
	vigorous	8 (8.1%)	45 (22.7%)	1	1
Soft drink	≥3x/week	77 (77.8%)	101 (51%)	3.36 (1.9,5.8)	3.7 (1.8, 7.3)**
	<3x/week	22 (22.2%)	97 (49%)	1	1
Sedentary behavior	≥3 hours	52 (52.5%)	25 (12.6%)	7.6 (4.3,13.6)	8.6 (4.3, 17)**
	<3 hours	47 (47.5%)	173 (87.4%)	1	1
Nutritional knowledge	Good	22 (22.2%)	78 (39.4%)	1	1
	Poor	77 (77.8%)	120(60.6%)	2.2 (1.3,3.9)	3.4 (1.7, 6.9)*

*P <0.05

**P<0.01

^^^COR: Crude Odd Ratio

^#^AOR: Adjusted Odd Ratio

## Discussion

The findings of this study showed that high wealth index, soft drink consumers more than or equal 3 times per week, physical inactivity, spending free time by watching movies (sedentary behavior) ≥3hours per day and poor nutritional knowledge were independent significant predictors of overweight and/or obesity.

In this study, adolescents from high socioeconomic families were more likely to be overweight than adolescents from low socioeconomic families. This finding was consistent with study findings from different developed countries including Russia and Italy [21, 22], and developing countries including South Africa and Ethiopia (Gondar and Hawassa) [6, 9, 23]. Similar findings from Pakistan showed an increased risk of being overweight and obesity was found in adolescents from wealthier families [24]. Diets are changing wherever incomes are rising in the developing world, with a marked shift from fruit and vegetables to meat, fats, and sugar [2]. This finding might be related to the diets, adolescents from higher socio-economic groups were well known to adopt western life leading to greater consumption of energy-dense foods which may substitute healthier local available diet like fruits, vegetables, cereals, etc. Moreover sedentary lifestyle because they are transported to and from school by car and bus might pose additional risks. Some studies from developed countries were not in line with the present findings. In Island, the level of overweight and obesity was higher in the lower socioeconomic groups [24]. In addition, the study finding from America was inconsistent with this finding. The possible reason for this discrepancy might be related to in developed countries, adolescents in higher socioeconomic groups tend to have a healthier diet [22], while in developing countries adolescents from high socio-economics are prone to energy-dense food consumption [23, 24]. This implies that high socioeconomic status may be attributable to the change in life style and dietary pattern that leads to obesity.

This study documented a significant association between physical inactivity and overweight/obesity which is similar to the results of the study conducted in Gondar [23] and Hawassa [9]. This finding was consistent with the finding from Pakistan that indicated that lack of physical activity was found to be significantly associated with overweight and obesity in children and adolescents [24]. Another result from the USA revealed that regular physical activity was an important factor in reducing the prevalence of overweight and obesity [22]. Additionally, WHO reported from Switzerland [15, 25] showed lack of physical activity had a positive association with overweight and obesity. This finding might be related to the lack of energy expenditure because of a lack of physical activity [15]. However, our finding is in contrast to the study in Iran and UAE [26, 27].

This study also found a significant positive association between sedentary behavior of ≥3hr/day and overweight/obesity, which is in agreement with the study conducted in Addis Ababa [7] and Jimma [8]. This study is consistent with studies done in Spain [28], Brazil [29], Munich, Germany [30], Tamale metropolis of Ghana [31], and Ghana and Uganda [32] that revealed that children who spent their free time viewing television, playing a computer game for 3 and more hours were more likely to be overweight and obese. This might be explained by the advancement in technology to change the lifestyle of children. Watching Television and playing a computer games may have decreased the amount of time spent on playing outdoor games which might have resulted in gaining extra weight. Sedentary behaviors were one of the risk factors for childhood overweight and obesity.

Evidence shows that a high intake of soft drinks positively correlates with obesity [33, 34]. It is believed that intake of soft drinks contributed greatly to weight gain by high added sugar content, low satiety, and incomplete compensation for total energy [35]. This study revealed that soft drink intake 3x/week and above was significantly associated with overweight and/or obesity compared to less than 3x/week. This finding was in line with findings from Ethiopia and Bangladesh and Brazil [13, 23, 36]. This could be explained as soft drinks and sweet food items are calorie-dense food which results in positive energy balance to their consumers.

In the present study, adolescents who had poor knowledge of nutrition were more prone to overweight and obesity than adolescents who had more knowledge of nutrition. This study, supported by a study conducted in six Latin America and Israel, suggests that the increased prevalence of overweight among adolescents is due to a deficit in nutritional knowledge in adolescents compared to those who had good knowledge in nutrition [37, 38]. In contrast, a study from Northern Ireland showed that knowledge deficit may not be the most significant factor preventing overweight individuals from adopting a healthier diet and questions the utility of purely educational approaches to dietary behavior change [39]. The difference might be related to the methodology that was adapted questionaries and overweight/obesity individuals were seeking out information of weight and advice.

The above result and discussion imply that preventing and reverting the progress from low energy food consumption, less screen time, and physically active state to energy-dense food consumption, longer screen time, and the physically inactive state plays a key role in reducing likely hood that adolescence becomes overweight and/or obese.

The limitation of this study was not addressed the genetic and health condition of participants. Even though different efforts were used to minimize recall bias and social desirability by using birthday or festivals for age and started with the nearest day for food frequency questionnaire, fast food, soft drinks, and physical activity still the result might be over or under-report. So, this might affect the estimation of the Odds Ratio. BMI for age was also wrongly misclassified the individual as overweight or obese while their body is built with muscle that increases the weight of the body.

## Conclusion

Even though the factors of overweight and obesity are complex and are not limited, the present study isolated certain socioeconomic, dietary, and lifestyle factors that are linked with greater risk of overweight and obesity among this population. Accordingly, high household wealth status, soft drink intake, being physically inactive, sedentary behavior, and poor knowledge in nutrition were significantly associated with overweight and obesity.

Adolescence provides a window of opportunity for the long-term positive impact that nutrition should be a programmatic priority in adolescents. Therefore based on our findings, we forward the following issues, school-based and parent promotion of healthy nutrition behaviors, limit sedentary behaviors, establish standards for school structured physical activity educations/sessions which are enjoyable, meet the needs, and develop a school nutrition club to promote adolescent’s nutritional knowledge and concur with physical activity and healthy diets among adolescents are the core elements to prevent overweight and obesity.

## Supporting information

S1 File English version questionnaire. (DOCX)

S2 File Amharic version questionnaire. (DOCX)

S3 File Data set of the study. (SAV)

S4 File Operational and term definition of risk factors for overweight and/or obesity. (DOCX)

S5 File Ethical clearance letter by IRB of Jimma University. (PDF)

## Abbreviations

BAZ: Body Mass Index for Age Z score
DDS: Dietary Diversity Score
GPAQ: Global Physical Activity Questioner
KMO: Kaiser-Meyer-Olkin
NCDs: Non-Communicable Diseases
TEM: Technical Error of Measurement
WDDS: Women Dietary Diversity Score
PAL: Physical Activity Level
IDDS: Individual Dietary Diversity Score
