# Supplementary material for: Determinants of overweight and/or obesity among school adolescents in Butajira Town, Southern Ethiopia. A case-control study
Source: PLoS One. 2022 Jun 28;17(6):e0270628. doi: 10.1371/journal.pone.0270628 (PMC9239474; doi:10.1371/journal.pone.0270628)
Supplement: S1 File — (DOCX) [file pone.0270628.s001.docx]

**ANNEXES**

**Annex I: Consent form**

**Written consent form for child parents and participant Information Sheet**

Good morning/ afternoon?

My name is _____________________. Currently I am a graduate student at Jimma University Institute of Health, Department of Population and Family Health. And now I am conducting a research on determining the predictors of overweight and obesity among school adolescent chi in Butajira town.

**Objective**: To determine the predictors of overweight and obesity among school adolescent in Butajira town, Gurage zone, SNNPR, Ethiopia 2019

You are selected randomly as a possible participant in this study as a subject.

**Potential risks**: There is no potential risk that may cause any harm on study participants.

**Benefits:** No financial benefits are related with this study. But by participating in this study, you contribute to improve the prevention and control method of adolescent overweight and obesity. **Confidentiality:** You and your child name will not be written in this form and will never be used in connection with any information you tell us. All information given by you and your child will be kept strictly confidential. Your participation is voluntary and you are not obligate to answer any question which you do not wish to answer. If you fill discomfort to respond to the questioner, please fill free to drop it. This questionnaire will take about 10 minutes.

**Written consent form for child parents**

I understand all about the objective and the process of the study. My and my child participation is voluntary and not obligate to answer any question which I do not know or do not wish to answer. I also understood that all information given by me and my child will be kept strictly confidential. Therefore, I am willing to participate in this study.

Study participant sign _____________________date_____________

Data collector sign ________________________date_____________

**Annex II: Questionnaires English Version**

This questionnaire is designed to collect information from respondents in respect to determine the predictors of the overweight and obesity among school adolescent in Butajira town.

**Part I: Questionnaire for sociodemographic surveillance to be filled by student Participant about their parent’s code______________**

| S , no | **Demographic information** | **Response** | | **skip** |
| --- | --- | --- | --- | --- |
| 101. | Religion | 1. | Muslim |  |
|  |  | 2. | Orthodox |  |
|  |  | 3. | catholic |  |
|  |  | 4. | protestant |  |
|  |  | 5. | other__________ |  |
| 102. | What is the occupation of the Mothers? | 1. | House wife |  |
|  |  | 2. | Government Employee |  |
|  |  | 3.  4. | Private Business  others |  |
| 103. | What is the occupation of the Mothers? |  | Government employee  Daily laborer  Private business  others |  |
| 104. | Ethnicity | 1. | Gurage |  |
|  |  | 2. | Amhara |  |
|  |  | 3. | Silte |  |
|  |  | 4. | Oromo |  |
|  |  | 5. | Tigre |  |
|  |  | 6. | other__________ |  |
| 105. | What is the highest level of maternal education you have completed? | 1.  2. | No formal education  1-8 grade |  |
|  |  | 3. | 9-12 grade |  |
|  |  | 4. | College/ university |  |
|  |  |  |  |  |
|  |  |  |  |  |
| 106. | What is the highest level of paternal education you have completed? | 1.  2.  3.  4. | No formal education  1-8 grade  9-12 grade  college/university |  |
| 107. | How many people live in your household? | _________________ | |  |

| **Household Wealth**  ***Now I will ask you about some fixed assets that your household have*.** | | | |
| --- | --- | --- | --- |
| Does the household have any of the following properties? **(Circle)** | | **Yes** | **No** |
| 108. | Functioning radio/Tape recorder/CD player | 1 | 0 |
| 109. | Functioning Television | 1 | 0 |
| 110. | Gas Stove | 1 | 0 |
| 111. | Electric stove | 1 | 0 |
| 112. | Bicycle | 1 | 0 |
| 113. | Motor Cycle | 1 | 0 |
| 114. | Cart/Gari | 1 | 0 |
| 115. | Bajaj | 1 | 0 |
| 116. | Mobile phone | 1 | 0 |
| 117. | Plough | 1 | 0 |
| 118. | Sofa | 1 | 0 |
| 119. | Spring mattress | 1 | 0 |
| 120. | Sponge/Foam mattress | 1 | 0 |
| 121. | Cotton mattress | 1 | 0 |
| 122. | Car | 1 | 0 |
| 123. | Generator | 1 | 0 |
| 124. | Milling | 1 | 0 |
| 125. | Water pump | 1 | 0 |

**Verbal assent form for students**

Good morning/ afternoon?

My name is _____________________. Currently I am a graduate student at Jimma University Institute of Health, Department of Population and Family Health. And now I am conducting a research on determining the predictors of overweight and obesity among school adolescent in Butajira town.

**Objective**: To determine the predictors of overweight and obesity among school adolescent in Butajira town, Gurage zone, SNNPR, Ethiopia 2019.

You are selected randomly as a possible participant in this study as a subject.

**Potential risks**: There is no potential risk that may cause any harm on study participants. **Benefits:** No financial benefits are related with this study. But by participating in this study, you contribute to improve the prevention and control method of adolescent overweight and obesity.

**Confidentiality:** Your name will not be written in this form and will never be used in connection with any information you tell us. All information given by you will be kept strictly confidential. Your participation is voluntary and you are not obligate to answer any question which you do not wish to answer. If you fill discomfort to respond to the questioner, please fill free to drop it. This questionnaire will take about 20 minutes.

Study participant sign _____________________date_____________

Interviewer sign ________________date_______________code_____________

Part-II; Questionnaire for Factor Surveillance for **Adolescent** students in Butajira Town.
Participant code ____________ Kebele------------- Name of the school ---------

| **1:Sociodemographic of the children, Location and type of school** | | | |
| --- | --- | --- | --- |
| **Question** | | **Response** | Skip rule |
| 126. | **Sex** | 1. Male 2. Female |  |
| 127. | Age | **_______________** |  |
| 128. | Type of school | 1. Government 2. Private |  |
| 129. | Grade | __________ |  |

**Dieting question**

| **For the following questions, please tell me how often in a week did you consume the following food items from the nearest day?** | | | | | | | | | |
| --- | --- | --- | --- | --- | --- | --- | --- | --- | --- |
| **s/no** | **Food item** | | | | | **Frequency per week** | | | |
|  |  |  |  |  |  | **Never** | **1-2 times** | **3-4 times** | **>4times** |
| 130. | Bread (“Furno”) | | | | |  |  |  |  |
| 131. | Teff (Injera) | | | | |  |  |  |  |
| 132. | Porridge (genfo) | | | F/m teff | |  |  |  |  |
|  |  |  |  | F/m wheat | |  |  |  |  |
| 133. | food made f/m maize | | | | |  |  |  |  |
| 134. | food made f/m barely | | | | |  |  |  |  |
| 135. | food made f/m sorghum | | | | |  |  |  |  |
| 136. | Wheat (Pasta, macaroni, Rice) | | | | |  |  |  |  |
| 137. | Bean, pea and lentils | | | | |  |  |  |  |
| 138. | Nuts | | | | |  |  |  |  |
| 139. | Banana, Mango, Avocado, | | | |  | |  |  |  |
| 140. | Papaya, Orange, Gishta | | | |  | |  |  |  |
| 141. | Butter | | | |  | |  |  |  |
| 142. | Oil | | | |  | |  |  |  |
| 143. | Milk, Cheese and yogurt | | | |  | |  |  |  |
| 144. | Meat | | Beef, Goat, Sheep/lamb | |  | |  |  |  |
|  |  |  | Chicken | |  | |  |  |  |
|  |  |  | Fish | |  | |  |  |  |
| 145. | chips, biscuit/sambusa, cookies | | | |  | |  |  |  |
| 146. | Egg | | | |  | |  |  |  |
| 147. | Sandwich made from vegetables | | | |  | |  |  |  |
| 148. | Sweet potato | | | |  | |  |  |  |
| 149. | Potato (White) | | | |  | |  |  |  |
| 150. | Carrot | | | |  | |  |  |  |
| 151. | Tomato | | | |  | |  |  |  |
| 152. | Keysir | | | |  | |  |  |  |
| 153. | Vegetable | “Salata” | | |  | |  |  |  |
|  |  | “Habesha gomen” | | |  | |  |  |  |
|  |  | Cabbage (teqel gomen | | |  | |  |  |  |

| 154. | Do you ever have a snack? | 1. Yes **2.** No | if no go to Q 156 |  |  |
| --- | --- | --- | --- | --- | --- |
| 155. | How many times a day do you have snack? | ________________ |  |  |  |
| 156. | How many meal do you have a day other than snacks? | ________________ |  |  |  |
| 157. | Do you eat out-side the home? | 0. no 1. yes |  |  |  |
| 158. | Do you have habit of missing any of your breakfast schedules? | 1. Yes 2. No |  | |  |
| 159. | Do you consume sweet food and soft drink | 1.yes  2. no |  | |  |
| 160. | How many servings of soft drink do you drink per week | ………..per week |  | |  |
| 161. | How many servings of soft drink do you drink per day | ………….per day |  | |  |
| **3: Physical Activity** | | |  | |  |
| **For the following questions, please tell me how often in a week did you physical exercise the following items from the nearest day?** | | | | | |
|  | **Question** | **Response** | **Skip rule** | | |
| 162. | Do you engage in Work besides your education? | 1. Yes 2. No | If no go to Q 169 | |  |
| 163. | If your answer in Q 162 is yes does your work involve vigorous intensity activity that for at least 10 minutes continuously? | 1. Yes 2. No | If no go to Q 166 | |  |
| 164. | In a typical week on how many days do you do vigorous –intensity activities as part of your work? | Number of days **_____** |  | |  |
| 165. | How much time do you spend doing vigorous – intensity activities at work on a typical day? | Hours: minutes **______** |  | |  |
| 166. | Does your work involve moderate intensity activity that for at least 10 minutes continuously? | 1. yes 2. No | If no go to Q 169 | |  |
| 167. | In a typical week on how many days do you do moderate –intensity activities as part of your work? | -------------------- |  | |  |
| 168. | How much time do you spend doing Moderate – intensity activities at work on a typical day? | Hours: minutes ------------------ |  | |  |
| **Travel to and from places, sports/recreation activities** | | | | |  |
| The next questions exclude the physical activities at work beside of your education that you have already mentioned. Now I would like to ask you about the usual way you travel to and from places. For example work, school, place of worship | | | | |  |
| 169. | Do you walk or use a bicycle for at least 10 minutes continuously to get to and from places? | 1. Yes 2. No | If no go to Q 172 | |  |
| 170. | In a typical week on how many days do you walk or use a bicycle for at least 10 minutes continuously | **________________** |  | |  |
| 171. | How much time do you spend walking or bicycling for travel in a typical day? | Hours: minutes __________ |  | |  |
| 172. | Mode of transportation | 1. foot  2 .vehicle |  | |  |

| 173. | Do you do any vigorous-intensity sports for at least 10 minutes continuously? | 1. Yes 2. No | If no go to Q 176 |
| --- | --- | --- | --- |
| 174. | If yes Q172, In a typical week on how many days do you do vigorous –intensity sports, fitness activities? | **________________** |  |
| 175. | How much time do you spend doing vigorous – intensity sports, fitness or recreational activities in a typical days? | Hours: minutes **___________** |  |
| 176. | Do you do any moderate-intensity sports for at least 10 minutes continuously? |  |  |
| 176. | In a typical week on how many days do you do moderate –intensity sports, fitness activities? | **_____________** |  |
| 177. | How much time do you spend doing moderate – intensity sports, fitness or recreational activities in a typical days? | Hours: minutes**_________** |  |
| **4:Sedentary behavior** | | | |
| 178. | How much time do you spent your free time by reading books, watching TV and video Playing on computer games per day | Hours……  Minute…… |  |
| 179. | Do you have sleeping habit in afternoon | 1. Yes 2. No |  |
| 180. | Average Sleep duration in particular day | Hours __________ |  |

| **6: Nutritional knowledge** | | |
| --- | --- | --- |
| I am going to ask you some questions about your nutrition and nutrition in general. Please let me know if you need me to clarify any of my questions. Feel free to ask any question you may have. | | |
| 181. | Do you know eating fruit and vegetables are prevents heart disease, diabetes, hypertension and cancer? | 1. Yes 2. No |
| 182. | Do you think people who are overweight are more likely to have health problems than people who are not overweight? | 1. Yes 2. No |
| 183. | Do you think people who are physically active are more likely to have health problems than people who are physically in-active? | 1. Yes 2. No |
| 184. | Do you think eating fruits and vegetables protects you from diseases? | 1. Yes 2. No |
| 185. | Do you know eating salty, sugar and fatty snacks every day leads to overweight and obesity? | 1. Yes 2. No |
| 186. | Do you know sticky and sugar-rich foods, such as sweets and candies causes interfere with appetite, can cause tooth decay and overweight and/or obesity | 1. Yes 2. No |
| 187. | Do you aware the bread, cereal, rice and pasta group is a good source of fat? | 1. Yes 2. No |
| 188. | Do you think Vitamin K is most important for healthy vision? | 1. Yes 2. No |
| 189. | Do you think iodine is most important for prevention of goiter? | 1. Yes 2. No |
| 190. | Do you think iron is most important for prevention of anemia? | 1. Yes 2. No |
| 191. | Do you think balanced diet is most preventive way of overweight and obesity? | 1.Yes 2. No |
| 192. | Do you know drinking alcohol causes interfere with appetite, can cause overweight and obesity? | 1.Yes 2. No |
| 193. | Do you aware eating fish meat is better than beef meat ? | 1.Yes 2. No |

**Part III: Questionnaire for Overweight and obesity and associated Factor Surveillance for
Physical Measurements to be filled by data collectors**

| **measurements** | **Response** | **Results** |
| --- | --- | --- |
| 194. | Height | In centimeters **_____________** |
| 195. | Weight | In kilograms (Kg) ________ |

Thank You for your cooperation!!
