# Supplementary material for: Determinants of overweight and/or obesity among school adolescents in Butajira Town, Southern Ethiopia. A case-control study
Source: PLoS One. 2022 Jun 28;17(6):e0270628. doi: 10.1371/journal.pone.0270628 (PMC9239474; doi:10.1371/journal.pone.0270628)
Supplement: S2 File — (DOCX) [file pone.0270628.s002.docx]

Amharic version of the questioner

ጅማ ዩኒቨርስቲ ጤና ኢኒስቲዩት የስነ-ህዝብ እና ጤና ትምህርት ክፍል ከጤና ጋር የተያያዘ ጥናታዊ ፅሁፍ የተሳታፊዎች መረጃ መስጫ ቅጽ

**እንደምን አደሩ/ዋሉ?**

--------------እባላለሁ፡፡በጅማ ዩኒቨርሲቲ ጤና ሳይንስ ኢንስቲትዩት ጤና ትምህርት ክፍል በስርዓተ-ምግብ የ2ኛ ዓመት የማስትሬት ድግሪ ተመራቂ ተማሪነኝ፡፡ በአሁኑ ሰዓት በቡታጅራ ከተማ እድሜየቸው ከአስር ዓመት እስከ አስራ-ዘጠኝ ዓመት ዕድሜ ለሚማሩ ተማሪዎች እና ወላጆችቻቸዉ ስለ ክብደት መጨመር እና ለክብደት መጨመር ተያያዥነት ያላቸዉን ነገሮች ለመለየት በማጥናት ላይ እገኛለሁ፡፡

**የጥናቱአላማ፡** የልጆች የክብደት መጨመር እና ለክብደት መጨመር ተያያዥነት ያላቸዉን
ነገሮች ለመለየት ነዉ፡፡

**የጎንዮሽጉዳት፡** በዚህ ጥናት መሳተፍ በርሶዎም ሆነ በልጅዎ ላይ ምንም አይነት ጉዳት አያመጣም፡፡

**ጥቅማጥቅም፡** በዚህ ጥናት መሳተፍ ምንም አይነት ገንዘብ አያስገኝም፡፡ ከአንተ\ች የምናገኘዉ መረጃ ከልጆች ክብደት መጨመር ጋር ተያይዘዉ የሚመጡ የልጆችን የጤና ችግሮች ለመከላከል፣ እቅድ ለማዉጣት ይረዳል። ስለዚህ ይህንን አስመልክቶ የተወሰኑ ጥያቄዎችን ልጠይቅዎት እወዳለሁ፡፡ የእርስዎ በእውነት ላይ የተመሰረተ መልስ ለዚህ ጥናት መሳካት አስተዋፅኦ ያደርጋል፡፡ እርስዎም የሚሰጡት መረጃ ከአጥኚውና ቃለመጠይቅ አድራጊው በስተቀር በማንኛውም መልኩ ለሌላ 3ኛ ወገን ተላልፎ አይሰጥም፡፡ በሙሉ ፈቃደኝት እንዲሳተፉ እየጠየቅሁ ያለመሳተፍ ወይም በማንኛውም ጊዜ ራስዎን ከጥናቱ የማግለል ሙሉ መብት አለዎት፡፡ በማንኛውም ጊዜ ጥያቄ ካለዎት በሚከተለው አድራሻዬ ማግኘት ይችላሉ፡፡ ጥያቄዎችን ለመመለስ አስር ደቂቃ ያህል ጊዜ ያስፈልጋል፡፡

የስምምነት መግለጫ ፎርም

ጅማ ዩኒቨርስቲ ጤና ኢኒስቲዩት የስነ-ህዝብ እና ጤና ትምህርት ክፍል በድህረ ምረቃ ፕሮግራም እኔ ስሜ ከዚህ በታች የተገለፀው፤ የዚህ ጥናት ዓላማ በደንብ የተብራራልኝ ሲሆን የጥናቱንም ዓላማ ተረድቻለሁ፡፡ በዚሁ ጥናት ላይ መሳተፍ በሙሉ ፈቃደኝነት ላይ የተመሰረተ መሆኑን በሚገባ የተረዳሁ ሲሆን በማንኛውም ጊዜ ከጥናቱ ራሴን የማግለል መብት እንዳለኝ አውቄአለሁ፡፡ ስለሆነም የምሰጠው መረጃ እስከ ተጠበቀ ድረስ በዚህ ጥናት ለመሳተፍ ተስማምቻለሁ፡፡ በዚህ ጥናት ለመሳተፍ ስምምነቴን ስገልፅ ለምጠየቀው ጥያቄ በእውነት ላይ የተመሰረተ መልስ ለመስጠት የተስማማሁ መሆኔን አረጋግጣለሁ፡፡

የመረጃ ሰጪው ፊርማ ___________________ ቀን _________________________
የአጥኚው ፊርማ _______________________ቀን _________________________

ለአሳዳጊ ቤተሰብ የስምምነት መግለጫ ፎርም

ጅማ ዩኒቨርስቲ ጤና ኢኒስቲዩት የስነ-ህዝብ እና ጤና ትምህርት ክፍል በድህረ ምረቃ ፕሮግራም እኔ ስሜ ከዚህ በታች የተገለፀው፤ የዚህ ጥናት ዓላማ በደንብ የተብራራልኝ ሲሆን የጥናቱንም ዓላማ ተረድቻለሁ፡፡ በዚሁ ጥናት ላይ ልጄ/ተማሪዬ እንዲሳተፍ በሙሉ ፍቃደኝነት ላይ የተመሰረትኩ መሆኑን በሚገባ ተረድቻለው፡፡ ስለሆነም ልጄ/ተማሪዬ የምሰጠው መረጃ እስከ ተጠበቀ ድረስ በዚህ ጥናት እንዲሳተፍ የተስማማሁ መሆኔን አረጋግጣለሁ፡፡

የመረጃ ሰጪው ፊርማ ___________________ ቀን _________________________
የአጥኚው ፊርማ _______________________ቀን _________________________

**ለልጆች ክብደት መጨመር ተያያዠነት ያላቸዉን ነገሮች ለመለየት በተማሪ ሚሞላ ቅጽ
የሚከተሉትን ጥያቄዎች በጥንቃቄ ከነበቡ በኋላ ለእንዳንዱ በተሰጠዉ መልስ መስጫ ቦታ መልሱን ይሙሉ::**

የተሳታፊ መለያ ቁጥር---------------------ክፍል **------------**የክፍል አይነት**------------የመንግስት/የግል… የት/ት ቤቱ ስም……..**

| ተ.ቁ. | ጥያቄ | መልስ | ዝለል |
| --- | --- | --- | --- |
| 101. | ሀይማኖት | . ኦርቶዶክስ 2. ሙስሊም 3. ካቶሊክ 4. ፕሮቴስታንት 5. ሌላካለይጠቀስ…………….. |  |
| 102. | የእናት ስራ ድርሻ | 1. የቤት እመቤት 2. የመንግስት ሰራተኛ 3. የግል ንግድ(ስራ) |  |
| 103 | የአበት ስራ ድርሻ | 1. የመንግስት ሰራተኛ 2. የግል ንግድ(ስራ)  3. የቀን ውሎ ስራ |  |
| 104. | ብሔረሰብ | 1. ሶማሌ 2. ኦሮሞ 3. አማራ 4. ጉራጌ 5. ትግሬ 6. ሌላካለይጠቀስ…………….. |  |
| 105. | የልጁ እናት የትምህርት ደረጃ | 1. መደበኛ ትምህርት አልተከታተሉም 2. ከ1 እስከ 8ኛክፍል 3. ከ9 እስከ 12ኛክፍል 4. ኮሌጅወይምዩኒቨርሲቲ ትምህርትአተናቅቄያለሁ |  |
| 106. | የልጁ አባት የትምህርት ደረጃ | 1. መደበኛ ትምህርት አልተከታተሉም 2. ከ1 እስከ 8ኛክፍል 3. ከ9 እስከ 12ኛክፍል 4. ኮሌጅወይምዩኒቨርሲቲ ትምህርትአተናቅቄያለሁ |  |
| 107. | እርሶዎን ጨምሮ የቤተሰብ ብዛት ስንት ነዉ | -------------------------- |  |

| **የቤት ንብረት ሁኔታ**  አሁን ስለ ቤተቹህ የንብረት እና እሴት ሁኔታ እጠይቃቹሃለሁኝ | | | |
| --- | --- | --- | --- |
| ንብረት ካለ አለን ክበብ ከሌለ ደግሞ ዬለምን ክበብ | | **አለ** | **ዬለም** |
| 108. | የሚሰራ ሬዲዮ/ቴፕ/ሲዲ አለ | 1 | 0 |
| 109. | የሚሰራ ቲቪ አለ | 1 | 0 |
| 110 | የጋዝ እስቶቭ አለ | 1 | 0 |
| 111. | የኤሌክተሪክ እስቶቭ አለ | 1 | 0 |
| 112. | በይስክል አለ | 1 | 0 |
| 113. | ሞተር ሰይክል አለ | 1 | 0 |
| 114. | ጋሪ አለ | 1 | 0 |
| 115. | ባጃጅ | 1 | 0 |
| 116. | የእጅ ስልክ አለ | 1 | 0 |
| 117 | መረሻ | 1 | 0 |
| 118. | ሶፋ አለ | 1 | 0 |
| 119. | ሚለጠጥ ፍራሽ አለ | 1 | 0 |
| 120. | የስፖንጅ ፍራሽ አለ | 1 | 0 |
| 121. | የጥጥ ፍራሽ አለ | 1 | 0 |
| 122. | መኪና | 1 | 0 |
| 123. | ጀነሬተር አለ | 1 | 0 |
| 124. | ወፍጮ አለ | 1 | 0 |
| 125. | የውሃ ምንጭ አለ | 1 | 0 |

| የተማሪዎች መግለጫ፣ የትምህርት ቤቱ አይነት | | | |
| --- | --- | --- | --- |
| ጥያቄ | | መልስ | ዝለል |
| 126. | ጾታ | 1. ወንድ 2. ሴት |  |
| 127. | እድሜ | --------------------- |  |
| 128. | የትምህርትደረጃ | --------------------- |  |
| 129. | የትምህርት ቤቱ አይነት | 1. የመንግስት 2. የግል 3. መንግስታዊ ያልሆነ ድርጅት 4.የመስጊድ/የቤተክርስቲያን/ካቶሊክ ትምህርት |  |

| **2: የአመጋገብ ልምድ ጋር የተየያዙ ጥያቄዎች**  ከዚህ ቀጥሎ ባለፈዉ አንድ ሳምንት አዘዉትረዉ ስለተመገቡአቸዉ የምግብ አይነቶች በተመለከተ እጠይቅዎታለሁ። | | | | | | | | | | | |
| --- | --- | --- | --- | --- | --- | --- | --- | --- | --- | --- | --- |
|  | **የምግብ አይነት** | | | | | | **በሳምንት ስንት ግዜ ይመገባሉ** | | | | |
|  |  |  |  |  |  |  | **አልተመገብኩም** | | **1-2 ጊዜ** | **3-4 ጊዜ** | **ከ4 ጊዜ በላይ** |
| 130. | የስንዴ ዳቦ(“ፉርኖ”) | | | | | |  | |  |  |  |
| 131. | የጤፍ እንጀራ | | | | | |  | |  |  |  |
| 132. | ገንፎ | | | | ከገብስ | |  | |  |  |  |
|  |  |  |  |  | ከስንዴ | |  | |  |  |  |
| 133. | ከቦቆሎ የተሰራ ምግብ | | | | | |  | |  |  |  |
| 134. | ከገብስ የተሰራ ምግብ | | | | | |  | |  |  |  |
| 135. | ከማሽላ የተሰራ ምግብ | | | | | |  | |  |  |  |
| 136. | ሩዝ፣ መኮሮኒ፣ ፓስታ | | | | | |  | |  |  |  |
| 137. | ባቄላ፣ አተር፣ ሽምብራ | | | | | |  | |  |  |  |
| 138. | ለውዝ | | | | | |  | |  |  |  |
| 139. | ሙዝ፣ አቨካዶ፣ መንጎ | | | | |  | |  | |  |  |
| 140. | ፓፓያ፣ ቡርትካን፣ ጊሽጣ | | | | |  | |  | |  |  |
| 141. | ቂቤ | | | | |  | |  | |  |  |
| 142. | ዘይት | | | | |  | |  | |  |  |
| 143. | ወተት፣ አይቤ፣ ኡርጎ | | | | |  | |  | |  |  |
| 144. | ስጋ | | የከብት/ፊያል/በግ | | |  | |  | |  |  |
|  |  |  | ዶሮ | | |  | |  | |  |  |
|  |  |  | ዓሳ | | |  | |  | |  |  |
| 145. | ቺፕስ፣ኩኪስ፣ብስኩት | | | | |  | |  | |  |  |
| 146. | እንቁላል | | | | |  | |  | |  |  |
| 147. | ሳንዲዊች | | | የኣትክልት | |  | |  | |  |  |
|  |  |  |  | የእንቁላል | |  |  |  |  |  |  |
| 148. | ስኳር ድንች | | | | |  | |  | |  |  |
| 149. | ድንች | | | | |  | |  | |  |  |
| 150. | ካሮት | | | | |  | |  | |  |  |
| 151. | ቲማቲም | | | | |  | |  | |  |  |
| 152. | ቀይስር | | | | |  | |  | |  |  |
| 153. | አትክልት | ሰላጣ/ቆስጣ/ፎሶሊያ | | | |  | |  | |  |  |
|  |  | ሀበሻ ጎማን | | | |  | |  | |  |  |
|  |  | ጥቅልል ጎመን | | | |  | |  | |  |  |

| 154. | በቁርስና በምሳ መካከል ወይም በምሳና በእራት መካከል ወይም ከመደበኛዉ አመጋገብ በተጨማሪ ምግብ ይመገባሉ? | 1. አዎ 2. አልመገብም: | መልሱ አልመገብም ከሆነ ወደ ጥያቄ 138 ይህዱ |
| --- | --- | --- | --- |

| 155. | በቀን ስንት ጊዜ መክሰስ ይመገባሉ? | ____________ |  |
| --- | --- | --- | --- |
| 156. | ከላይ የጠቀሱትን ምግብ ሳይጨምር በቀን ሰንት ጊዜ ይመገባሉ? | ____________ |  |
| 157. | ከቤት ውጭ ይመገባሉን | 1 አዎ  2 አልመገብም |  |
| 158. | አንድ አንድ ቀን ቁርስ፤ ሳይበሉ የሚቀሩበት ቀን አለ? | 1. አዎ 2. የለም |  |
| 159. | ለስላሳ የመጠጣት ልምድ አሎት | 1. አዎ  2. የለም |  |
| 160. | አብዛኛዉን ጊዜ በአንድ ሳምንት ውስጥ ስንት ቀን ለስላሳ መጠጦች ይጠጣሉ? ለምሳሌ ኮካ፣ ፕፐሲ እናም ሌሎችም? | ቀን………….. |  |
| 161. | ለስላሳ መጠጦች ከሚመገቡባችዉ ቀናት ዉስጥ በአንዱ ቀን ስንት ጊዜ ይጠጣሉ? | ብዛት………. |  |

| አካላዊ እንቅስቃሴ እና ስራ ጋር የተየያዘ እንቅስቃሴ | | | |
| --- | --- | --- | --- |
| ክዚህ ቀጥሎ የተለያዩ የአካል እንቀስቃሴ በማካሄድ የሚያሳልፉአቸዉን ጊዜ በተመለከተ እጠይቅዎታለሁ። | | | |
| ጥያቄ | | መልስ | ዝለል |
| 162. | ከትምህርት ዉጭ ተጨማሪ የሚሰሩት ስራ አለ? | 1. አዎ 2. የለም | መልሱ የለም ከሆነ ወደ ጥያቄ 164 ይህዱ |
| 163. | ከዚህ በላይ ለተጠቀሰዉ ጥያቄ መልሰዎ አዎ ከሆነ ስራዎ ብርቱ ጉልበት የሚጠይቅ ተግባር ወይም ቶሎቶሎ መተንፈስን ወይም ፈጣን የልብ ምት ሊያስከትል የሚችል ተገባር ያለማቋረጥ ቢያንስ ለ10 ደቂቃ ይሰራሉ? | 1. አዎ 2. የለም | መልሱ የለም ከሆነ ወደ ጥያቄ 169 ይህዱ |
| 164. | አብዛኛዉን ጊዜ በሳምንት ስንት ቀን ብርቱ ጉልበት የሚጠይቅ ተግባር ያከናዉናሉ? | የቀን ብዛት __________ |  |

| 165. | ብርቱ ጉልበት የሚጠይቁትን ተግባራት ከሚያከናዉኑባቸዉ ቀናት በቀን ለምን ያህል ጊዜ ይሰራሉ? | ስዓት____ ደቂቃ ______ |  |
| --- | --- | --- | --- |
| 166. | ስራዎ መጠነኛ ጉልበት የሚጠይቅ ተግባር ወይም መጠነኛ የመተንፈስ ወይም የልብ ምት ፍጥነት ጭማሪ ሊያስከትል የሚችል ተገባር ያለቋረጥ ቢያንስ ለ10 ደቂቃ ይሰራሉ? | 1. አዎ 2. የለም | መልሱ የለም ከሆነ ወደ ጥያቄ 169 ይህዱ |
| 167. | አብዛኛዉን ጊዜ መጠነኛ ጉለበት የሚጠይቁ ስራዎችን በሳምንት ስንት ቀን ይሰራሉ? | የቀንብዛት__________ |  |
| 168. | መጠነኛ ጉልበት የሚጠይቁ ተግባራት ከሚያከናዉኑባቸዉ ቀናት በአንዱ ቀን ለምን ያህለ ጊዜ ይሰራሉ? | ስዓት____ ደቂቃ ______ |  |
| **ከዚህ ቀጥሎ ከቦታ ቦታ ሲንቀሳቀሱ በብዛት የሚጠቀሙባቸዉን መንገዶች እጠይቅዎታለሁ።** | | | |
| 169. | ከቦታ ቦታ በሚንቀሳቀሱበት ጊዜ ለ 10 ደቂቃ ያለማቋረጥ በእግረዎ ወይም በብስክሌት ይሄዳሉ። | 1. አዎ 2. አልሄድም | መልሱ አልሄድም ከሆነ ወደ ጥያቄ 172 ይህዱ |
| 170. | በሳምንት ዉስጥ ስንት ቀን ከ 10 ደቂቃ ያላነሰ ያለማቋረጥ በእግረዎ ወይም በብስክሌት ይሄዳሉ። | የቀን ብዛት______ |  |
| 171. | በነዚህ ቀናት ዉስጥ በቀን ምን ያህል ሰዓት ሳያቋርጡ በእግር ወይም የብስክሌት ጉዞ ያደርጋሉ? | ሰአት---------ደቂቃ------ |  |
| **ከስፖርትና ከመዝናናት ጋር የተየያዙ እንቅስቃሴዎች** | | | |
| 172. | ከፍተኛ የልብ ምት ወይም የአተነፋፈስ ፍጥነት መጨመር ሊያሰከትል የሚችል ስፖርት ቢያንስ ለ10 ደቂቃ ያክል ሳያቋርጡ ይሰራሉ? | 1. አዎ 2. የለም | መልሱ የለም ከሆነ ወደ ጥያቄ 175 ይህዱ |
| 173. | አበዛኛዉን ጊዜ በሳምንት ስንት ቀን ከፍተኛ የልብ ምት ወይም የአተነፋፈስ ፍጥነት መጨመር ሊያሰከትል የሚችል ስፖርት በያንስ ለ10 ደቂቃ ያክል ያለማቀዋረጥ ይሰራሉ? | የቀንብዛት________ |  |
| 174. | ስፖርት ከሚሰሩባቸዉ ቀናት ዉስጥ በአንዱቀን ሳያቋርጡ ለምን ያክል ጊዜ ከፍተኛ የልብ ምት ወይም የአተነፋፈስ ፍጥነት መጨመር ሊያሰከትል የሚችል ስፖርት ይሰራሉ? | ስአት________ደቂቃ_____ __ |  |
| 175. | መጠነኛ የሆነ የልብ ምት ወይም የአተነፋፈስ ፍጥነት መጨመር ሊያሰከትል የሚችል ስፖርት በያንስ ለ10 ደቂቃ ያክል ሳያቋርጡ ይሰራሉ? | 1. አዎ 2. የለም | መልሱ የለም ከሆነ ወደ ጥያቄ 172 ይህዱ |

| 176. | አበዛኛዉን ጊዜ በሳምንት ስንት ቀን መጠነኛ የሆነ የልብ ምት ወይም ያተነፋፈስ ፍጥነት መጨመር ሊያሰከትል የሚችል ስፖርት በያንስ ለ10 ደቂቃ ያክል ሳያቋርጡ ይሰራሉ? | የቀን ብዛት_____ |  |
| --- | --- | --- | --- |
| 177. | የእረፍት ስአትዎን ቴሌቭዥን፣ቭዲዮ በማየት እና ኮምፑተር ጌም በመጫዎት በቀን ለምን ያህል ሰዓት ያሳልፋሉ? | 1. ሰዓት………… 2. ደቂቃ……….. |  |
| 178. | ስፖርት ከሚሰሩባቸዉ ቀናት ዉስጥ በአንዱ ቀን ሳያቁርጡ ለምን ያህል ጊዜ መጠነኛ የሆነ የልብ ምት ወይም የአተነፋፈስ ፍጥነት መጨመር ሊያሰከትል የሚችል ስፖርት ሳያቋርጡ ይሰራሉ? | ሰአት________ደቂቃ_____ |  |
| 179. | በአብዛሃኛዉ ጌዜ ከሰአት በኋላ የመተኛት ልምድ አለህ/ሽ? | 1. አዎ 2. የለኝም |  |
| 180. | በአብዛሃኛዉ በአንድ ቀን ለስንት ሰአት በእንቅልፍ ተኝተዉ ያሳልፋሉ? | ሰአት__________ |  |

**ከዚህ ቀጥሎ ስለ ምግብ አጠቃቀም እና ጥቅሞች ላይ ጠቅላላ ዕውቀት እጠይቅዎታለሁ።**

| 181. | አትክልትና ፍራፍሬ መመገብ ደምግፊት፣የስኳር እና የልብ ህመምን እንደሚከላከል ያውቃሉ | 1. አውቀለሁ 2. አላቅም |
| --- | --- | --- |
| 182. | ከሚገበው በላይ የወፈረ ሰው ትክክለኛ ክብደት ከለው ሰው ይልቅ ለተለያዩ በሽታዎች እንደሚጋለጡ ያውቃሉ | 1. አውቀለሁ 2. አላቅም |
| 183. | የኣካል እንቅስቃሴ ማያደርጉ ሰዎች ለበሽታ የበለጠ ተጠቂ መሆናቸውን የውቃሉ | 1. አውቀለሁ 2. አላቅም |
| 184. | አትክልትና ፍራፍሬ መመገብ በሽታን እንደሚከላከል ያውቃሉ | 1. አውቀለሁ 2. አላቅም |
| 185. | ጨው፣ሱኳርእና ስብ የበዛባቸው ምግቦች አዘውትሮ መመገብ ላልተገባ ክብደት መጨመር ምክኒያት መሆኑን ያውቃሉ | 1. አውቀለሁ 2. አላቅም |
| 186. | ከረሜላ እና ስኳር ነክ ያላቸው ምግቦችን ስንመገብ የምግብ ፍላጎታችን እንደሚዛባ ያውቃሉ | 1. አውቀለሁ 2. አላቅም |
| 187. | ዳቦ፣ፓስታ እና ሩዝ ስብን እንደሚይዝ ያውቃሉ | 1. አውቀለሁ 2. አላቅም |
| 188. | ቫይታሚን ኤ የያዙ ምግቦች ለአይን እይታ ትልቅ ፋይዳ እንዳላቸው ያውቃሉ | 1. አውቀለሁ 2. አላቅም |
| 189. | አዮዲን የእንቅርት በሽታ ወይም ጎይተርን እንደሚከላከል ያውቃሉ | 1. አውቀለሁ 2. አላቅም |
| 190. | አይረን የደም ማነስን እንደሚከላከል ያውቃሉ | 1. አውቀለሁ 2. አላቅም |
| 191. | ጤናማ/የተመጣጠነ ምግብ የልተፈለገ ክብደት መጨመርን እንደሚቀንስ የውቃሉን | 1. አውቀለሁ 2. አላቅም |
| 192. | አልክሆል መጠጣት የምግብ ፍላጎትን እንዲሁም የልተፈለገ ክብደትን ለመጨመር ምክኒያት መሆኑን ያውቃሉ | 1. አውቀለሁ 2. አላቅም |
| 193. | የዓሳ ስጋ ከከብት ስጋ ያልተፈለገ ክብደትን ለመቀነስ የተሸለ መሆኑን ያውቃሉ | 1. አውቀለሁ 2. አላቅም |

በመረጃ ሰብሳቢዉ የሚሞላ አካላዊ ልኬት

| ልኬት | መልስ | ዝለል |
| --- | --- | --- |
| 194. | ቁመት | በሴንቲ ሜትር**_________** |
| 195. | ክብደት | በኪሎግራም________ |

ለተደረገልኝ ትብብር በጣም አድርጌ አመሰግናለሁኝ!!
