# Supplementary material for: Determinants of overweight and/or obesity among school adolescents in Butajira Town, Southern Ethiopia. A case-control study
Source: PLoS One. 2022 Jun 28;17(6):e0270628. doi: 10.1371/journal.pone.0270628 (PMC9239474; doi:10.1371/journal.pone.0270628)
Supplement: S4 File — (DOCX) [file pone.0270628.s004.docx]

### **Operational and Term definition**

**Thinness**: BMI-for –age Z scores ≤ −2SD [19, 20].

**Normal weight**: BMI-for –age Z scores between −2SD < BAZ ≤ +1SD [19, 20].

**Overweight**: BMI-for-age Z scores between +1SD < BAZ ≤ +2SD [19, 20]..

**Obese**: BMI-for-age Z scores above > +2SD [19, 20].

**Overweight and/or obesity**: BMI-for-age Z scores above >1SD [19, 20].

**High DDS-** is dietary diversity score of Consuming 5 & more food groups [18].

**Low DDS**- is dietary diversity score of Consuming 4 and less food groups [18].

**Snacking:** foods that are eaten between regular meals

**Skipping breakfast:** individual will be considered as skipping breakfast if she/he skips 3 or more breakfast per week

**Fast food:**  consumption of food made fast and calorie dense like burger, pizza, bombolinno, sanbusa per week [13].

**Physical activity:** the total time spent in physical activity during a typical week (total physical activity level); **Inactive**: inactive at work, transport and leisure time or less than 600 Metabolic Equivalent Task (MET) - minutes/week; **Moderate**: having a moderate activity or at least 600 MET-minutes per week and **Vigorous**: vigorous activity at work, transport or leisure time or at least 1,500 MET-minutes/weeks [15].

**Sedentary behaviors:** Time spent using Reading books, and or Watching TV, and or video playing on computer games more than 2hrs/day or less than 8 hours sleeps [12]

**Meal frequency:** number of regular meal intake 3 times per day

**Soft drinks:** Frequency of drinking sweetened beverages like mirinda, Coca-Cola, Fanta and others more than once per day [13].

**Eating outside:** eating the purchase and consumption of meals and snacks prepared outside the home.

**Dietary habit**: - Dietary habit refers to the number of days in which particular food group (e.g., cereals, flesh meat, fruits, vegetables, and meat, milk and milk products and others) that participants consumed in the past weeks at the time of data collection.

**Wealth index:** Socioeconomic index was developed as follows: first all study participants were asked about the ownership of fixed assets by their household with a score 1 given to those who own the asset and score of “0” given to those who did not own. Then principal component analysis was used to develop the wealth index and categorize in to 3 tertiles [11].

**Nutritional Knowledge Index**: Thirteen questionnaires were adapted and checked their reliability with a Cronbach’s alpha value of 0.73 from the Food and Agriculture Organization of United Nations for school children. Based on this question, the nutritional knowledge index variable was created using principal component analysis and categorized into two groups based on mean value. Thus, the first component students scored “poor knowledge” and the second component students scored “good knowledge” [14]**.**

**Notice:** you get the reference directly from the Manuscript.
