# Supplementary material for: Determinants of overweight and/or obesity among school adolescents in Butajira Town, Southern Ethiopia. A case-control study
Source: PLoS One. 2022 Jun 28;17(6):e0270628. doi: 10.1371/journal.pone.0270628 (PMC9239474; doi:10.1371/journal.pone.0270628)
Supplement: S5 File — (PDF) [file pone.0270628.s005.pdf]

JIMMA UNIVERSITY
ጅማ ዩኒቨርሲቲ

ቁጥር
Ref. No JHRPGD/690/19
ቀን
Date 19/02/2019

Institutional Review Board (IRB)
Institute of Health
Jimma University
Tel: +251471120945
E-mail: zeleke.mekonnen@ju.edu.et

To: Shemsu Kedir

Subject: Ethical approval of research protocol

The IRB of institute of health has reviewed your research project entitled:

"Determinants of over-nutrition (overweight and obesity) among school adolescent students in Buitajira Town: A case control study design

This is to notify that this research protocol as presented to the IRB meets the ethical and scientific standards outlined in national and international guidelines. Hence, we are pleased to inform you that your protocol is ethically cleared.

We strongly recommended that any significant deviation from the methodological details indicated in the approved protocol must be communicated to the IRB before they are implemented.

With regards!

Zeleke Mekonnen (PhD)
Associate Professor, Health Research and Postgraduate Director

Institute of Health Jimma University

Scanned by CamScanner
